# Efficacy of Injectable Bone Fillers for Alveolar Ridge Preservation: A Histomorphometrical Analysis

**DOI:** 10.1111/jcpe.14162

**Published:** 2025-04-10

**Authors:** Frank Schwarz, Ausra Ramanauskaite, Karina Obreja, Jonas Lorenz, Robert Sader, Puria Parvini

**Affiliations:** ^1^ Department of Oral Surgery and Implantology Goethe University, ZZMK Carolinum Frankfurt Germany; ^2^ Department for Oral, Cranio‐Maxillofacial and Facial Plastic Surgery Goethe University, University Hospital Frankfurt Frankfurt Germany

**Keywords:** alveolar ridge preservation, histological analysis, pre‐clinical study

## Abstract

**Aim:**

To assess the efficacy of injectable bone fillers for alveolar ridge preservation (ARP).

**Materials and Methods:**

The mandibular premolars (P2, P3, P4) were bilaterally assigned to ARP in a total of *n* = 9 beagle dogs. Each P was decapitated and hemisected under preservation of the mesial (i.e., devitalization and filling with calcium hydroxide) and removal of the distal root. The resulting 6 extraction sockets were randomly allocated to a total of four injectable test materials (i.e., bovine bone particles + porcine collagen, lyophilized materials reconstituted with either blood or saline [T1, T2, T4]; or ready‐to‐use wet material [T3]) and one control material (collagenated bovine bone mineral) (C) as well as one negative control group (N). Primary wound closure was ensured in all test and C groups. At 12 weeks, dissected blocks were prepared for histomorphometrical analyses. Buccal bone height (BBH) was defined as a primary outcome. Lingual bone height (LBH), buccal and lingual bone wall width (BBW and LBW 1, 3 and 5 mm infracrestally), surface area of bone and particles, fibrous tissues, and bone marrows were defined as secondary outcomes. Between‐group comparisons were assessed using ANOVA and Mann–Whitney tests.

**Results:**

After 12 weeks, all groups were associated with similar BBH values (BBH: 14.1, 14.0, 13.7, 13.8, 14.3 and 114.2 mm in the T1, T2, T3, T4, C and N groups, respectively; *p* > 0.05 for all between‐group comparisons). The BBW and LBW measurements were comparable among the groups. ARP sites showed a trend towards higher area measurements of bone and particle surfaces compared with the N group (11.5, 11.6, 13.1, 12.5, 9.1 mm^2^ in T1, T2, T3, T4, C and N groups, respectively). C, T1, T2, T3 and T4 particles were associated with a similar marked grade of osteoconduction and osteointegration within the former extraction socket area.

**Conclusions:**

Within its limitations, the present study has pointed to the similar efficacy of injectable bone fillers for ARP compared with the particulated bone substitute and negative control.

## 1 Introduction

Following tooth extraction, the alveolar ridge undergoes marked dimensional alterations leading to the reduction of the overall ridge volume and change in the ridge shape (Hammerle, Araujo, and Simion 2012). In the presence of a bone loss at the time of extraction (e.g., due to periodontitis), an even more pronounced reduction in tissue volume can be expected (Aimetti et al. 2018; Ramanauskaite et al. 2020). The resulting alveolar ridge contour has been proven to increase the need for bone augmentation procedures to either enable implant‐supported restorations (Avila‐Ortiz et al. 2019) or to improve the stability of removable dentures (Rignon‐Bret et al. 2022) as well as the aesthetic appearance of pontics along with conventional bridges (Song et al. 2022).

To limit the dimensional alterations following tooth extraction, the concept of alveolar ridge preservation (ARP) has been established (Hammerle, Araujo, and Simion 2012). ARP prior to dental implant placement was first documented by Artzi and Nemcovsky in 1998 and defined as the process of ‘maintaining the ridge volume within the confines of the existing envelope at the time of extraction’ (Artzi and Nemcovsky 1998). ARP treatment modalities have proven to be associated with a clinically relevant prevention of alveolar bone resorption when compared with untreated control sites (horizontal: 1.5–2.4 mm; vertical mid‐buccal: 1–2.5 mm; vertical mid‐lingual: 0.8–1.5 mm) (Tonetti et al. 2019). These outcomes were achieved by a variety of particulated bone grafts (e.g., bovine‐ and porcine‐derived xenografts, allografts, alloplastic material) with or without the socket sealing technique (Avila‐Ortiz et al. 2019). Although no superior ARP approaches could be determined, the application of particulated xenogenic or allogenic materials covered with an absorbable collagen membrane or rapidly absorbable collagen sponge was shown to be associated with the most favourable outcomes in terms of the preservation of horizontal ridge dimensions (Avila‐Ortiz et al. 2019). More recently, biological agents such as enamel matrix derivatives and leukocyte‐ and platelet‐rich fibrin have been incorporated alongside particulate bone fillers to accelerate the maturation of bone following alveolar ridge preservation (De Angelis et al. 2019; Lee et al. 2020). While enhanced soft‐tissue wound healing and reduced postoperative discomfort were observed, their impact on maintaining hard‐tissue dimensions proved to be minimal (De Angelis et al. 2019; Lee et al. 2020).

Previous experimental studies have shown that the handling of particulate augmentation materials, along with the compressive forces applied to the crestal surface of an extraction socket, directly influences the defect fill in the apical region. This, in turn, may limit new bone formation (Dahl et al. 2024; Delgado‐Ruiz et al. 2018). The aforementioned outcomes of ARP might be further improved by the usage of injectable and flowable bone fillers, which may allow for easier clinical handling of the material that is not dependent on the application pressure, resulting in subsequently more homogeneous grafting of all compartments of the extraction socket.

The aim of this pre‐clinical study was to evaluate the efficacy (i.e., dimensional alveolar ridge preservation) of four novel experimental injectable bone fillers applied into the fresh extraction sockets in an established canine model.

## 2 Materials and Methods

### 2.1 Animals

A total of *n* = 9 male beagle dogs (Breed: HsdRcc:DOBE) (Marshall, France) (aged at least 12 months, bodyweight range between 9.8 and 13.4 kg) were used.

Husbandry, housing and environment conditions conformed to the European requirements (Directive EU/2010/63). In particular, the dogs were housed in cages identified by a card indicating the study number, animal numbers, sex, and dates of beginning and end of the experimental in‐life phase. The animals were kept under laboratory conditions. Room temperature and relative humidity were recorded daily. The light cycle was controlled using an automatic timer (12 h of light, 12 h of dark).

A commercially available diet (SAFE, France) was provided twice daily. The dogs were acclimated to a soft diet before surgery and fed only a soft diet after surgery. Potable water was delivered *ad libitum* through species‐appropriate containers or delivered through an automatic watering system.

All surgeries were conducted at NAMSA (Chasse‐sur‐Rhône, France), an AAALAC international accredited facility, registered with the French Department of Agriculture for animal housing, care and investigations. The study protocol (APAFIS#12878‐201801021136883) was approved by the NAMSA Ethics Committee and considered the 3R (Replace, Reduce, Refine) guidelines for animal experimentation. The following reporting adhered to the ARRIVE Guidelines 2.0 for relevant items (Percie du Sert et al. 2020).

### 2.2 Study Design

In all animals, the mandibular premolars (P2, P3, P4) were bilaterally assigned to ARP and spontaneous healing. In particular, following hemisection and extraction, the resulting six extraction sockets were randomly allocated to a total of four test and one control materials (Geistlich, Wolhusen, Switzerland) for ARP, as well as one negative control group (i.e., spontaneous healing) (N) based on a computer‐generated code.

The following test groups were defined according to the use of different experimental injectable prototype bone filler materials:

- Test 1 (T1): lyophilized bovine bone particles 0.12–0.18 mm + porcine collagen (Geistlich) reconstituted with blood;
- Test 2 (T2): lyophilized bovine bone particles 0.12–0.18 mm + porcine collagen (Geistlich) reconstituted with saline;
- Test 3 (T3): a ready‐to‐use wet material (bovine bone particles 0.12–0.18 mm + porcine collagen) (Geistlich);
- Test 4 (T4): lyophilized bovine bone particles 0.12–0.18 mm + porcine collagen, (Geistlich) reconstituted with saline;
- Control (C): a collagenated bovine bone mineral (BioOss Collagen, Geistlich) moistened with saline.

Accordingly, each test and control treatment was allocated to a total of 9 extraction sites (*n* = 9 animals) (Table S1).

### 2.3 Pre‐Operative Procedure and Anaesthesia Protocol

Before their arrival at NAMSA, a full‐mouth scaling was performed by the breeder in all dogs. Spiramycin and metronidazole (Stomorgyl, Merial) were administered per os once a day for two days before surgery for antibiotic prophylaxis.

On the day of surgery, pre‐medication was performed by intramuscular injection of medetomidine (Dorbene Vet, Zoetis, NJ, USA) and buprenorphine (Buprecare, Axience, Pantin, France). Anaesthesia was induced by intravenous injection of ketamine (Ketamine 1000, Virbac, Carros, France). Each dog was intubated, mechanically ventilated and placed on isoflurane inhalant anaesthetic (IsoFlo, Zoetis) for continued general anaesthesia. An intravenous infusion with suitable electrolyte solution (Ringer lactate, Baxter, Deerfield, Il, USA) was performed during surgery. A pre‐operative subcutaneous injection of an anti‐inflammatory drug (carprofen, Rimadyl, Zoetis) was provided. A neutral ophthalmic ointment (Ocrygel, TVM, Berlin, Germany) was applied to both eyes to protect the corneas from drying and was re‐applied as needed. Lidocaine with adrenaline (Aguettant, Langenfeld, Germany) was administered bilaterally at each tooth site. The amount given was at the surgeon's discretion.

The dogs were placed in the lateral position on a warmed pad. A rectal temperature probe was placed during surgery. Electrocardiogram, peripheral non‐invasive arterial blood pressure and oxygen saturation were monitored. When any abnormality was detected, the appropriate clinical measures were taken, such as the intravenous injection of propofol (Propovet, Zoetis) in case of wake‐up signs, and intravenous injection of atropine (Aguettant) in case of bradycardia. At the end of the surgery, some animals had received an intramuscular injection of atipamezole (Alzane, Zoetis) if required by their clinical status.

### 2.4 Sample Size Calculation

A sample size calculation was not feasible due to insufficient reference data on these ARP protocols in the existing literature.

### 2.5 Surgical Procedures

**FIGURE 1:**
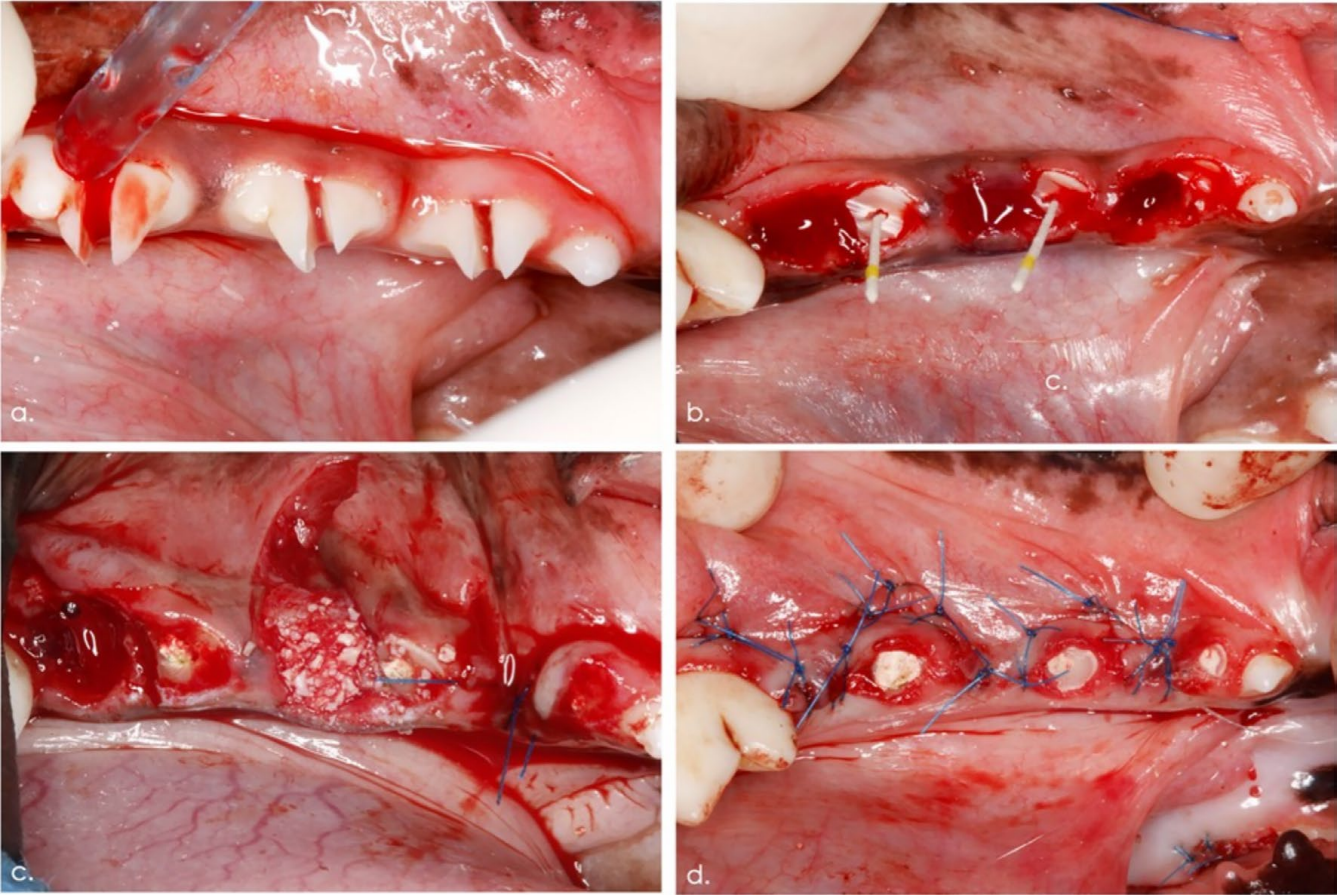
Surgical procedures. (a) Situation following careful hemisection under preservation of the inter‐radicular bone. (b) The distal root was removed and the mesial root was prepared for a provisional endodontic treatment (i.e., pulp extirpation, root canal enlargement, filling using calcium hydroxide paste, sealing using a self‐curing glass‐ionomer cement material). (c) The distal alveolar socket was completely filled by loosely applying the respective test and control materials. Negative control sites were left untreated and ensured to be filled with blood. (d) A tension‐free wound closure at the experimental sites was obtained by means of buccally elevated mucosal flaps.

Circumferential marginal incisions were made at each premolar (P2, P3 and P4). Subsequently, each tooth was hemisected using a fissure burr under saline irrigation along a bucco‐lingual axis. Particular care was taken to preserve the inter‐radicular crestal bone (Figure 1a).

Afterwards, a syndesmotome (Chompret, Stoma, Emmingen‐Liptingen, Germany) was superficially inserted between the distal root of the tooth and the alveolar bone, and a luxator blade (Luxelevator, Stoma) was pushed in an apical direction with controlled force and rotated around the distal root until it became loose in the alveolus. Small forceps (Davier Atlas, Hu‐Friedy, Frankfurt, Germany) were then used to remove the distal root by applying rotational and apical force. The alveolus was then rinsed with saline solution to remove potential debris. An elevator (Luxelevator, Stoma) and/or a bur were used to remove any remaining broken root fragments.

The pulp cavity of the mesial root was opened with an appropriate sharp ball burr and the pulp tissue extirpated with a sterile barbed broach (VDW, Munich, Germany). The root canal was progressively enlarged with size‐increasing root canal files (VDW) and rinsed with 0.9% NaCl solution (Baxter, Unterschleißheim, Germany). Subsequently, the root canal was dried with blunt paper points (Rotate, VDW) and filled using calcium hydroxide paste (Calasept Plus, Nordiska Dental, Angelholm, Sweden), while the coronal portion of the pulp chamber was sealed using a self‐curing glass‐ionomer cement material (CavitW, 3 M ESPE, Neuss, Germany) (Figure 1b).

Each distal alveolar socket was completely filled by loosely applying the respective test and control materials. An overfilling beyond the marginal bone levels was avoided. At the negative control sites, particular care was taken that the sockets were entirely filled with blood.

Subsequently, mucosal flaps were elevated at the buccal aspects and advanced by means of one vertical releasing incision to obtain a tension‐free wound closure at the experimental sites (i.e., distal extraction sites) (Figure 1c,d). Finally, the maxillary P2, P3 and P4 teeth were filed down without penetration of the pulp chamber to avoid any trauma to the experimental sites.

### 2.6 Postoperative Care

Each animal was moved to a recovery area and monitored for recovery from the anaesthetic until sternal recumbency was achieved. After recovery, each animal was returned to its cage and observed for general health. Postoperative antimicrobial prophylaxis (spiramycine and metronidazole) was administered *per os* after the surgery and daily for a period of 16–17 days. Carprofen (Carprodyl, Ceva) was provided daily *per os* for 6 days. The analgesic medication included a subcutaneous injection of buprenorphine (Buprecare, Axience) at the end of the surgery day, and twice a day for two days after the surgery.

Local disinfection included a daily application of a chlorhexidine solution until suture removal was performed after about 2 weeks.

### 2.7 Retrieval of Specimens

The animals were terminated at 12 weeks after surgery.

In particular, each animal was anaesthetised by an intramuscular injection of tiletamine zolazepam (Zoletil, Virbac) and received a subcutaneous injection of buprenorphine (Buprecare, Axience). Following intubation, they were mechanically ventilated and placed on isoflurane inhalant anaesthetic (lsoFlo, Zoetis) for continued general anaesthesia. Catheters were then inserted in the carotid arteries. All animals were heparinised (300 IU/kg, intravascular) (Heparin Choay, Sanofi‐Aventis, Paris, France). Subsequently, the dogs were euthanised by an injection of a lethal solution of pentobarbital (Dolethal, Vetoquinol). Post‐mortem perfusion was accomplished using 10% neutral buffered formalin.

### 2.8 Histological Preparation

Dissected blocks were immersed in 10% neutral buffered formalin, dehydrated using ascending grades of alcohol, cleared in xylene and embedded in polymethylmetacrylate (PMMA) for non‐decalcified sectioning. From each experimental site, two central bucco‐lingual cross sections were obtained by a laser microcutting system (approximately 10 μm thickness). Laser microcutting was conducted at LLS ROWIAK LaserLabSolutions GmbH (Hannover, Germany). All sections were stained with McNeal tetrachrome to perform qualitative and quantitative histomorphometric analyses.

### 2.9 Histomorphometrical Analysis

The histomorphometric analysis was conducted by a certified and calibrated pathologist at NAMSA according to the ISO 10993 standard.

**FIGURE 2:**
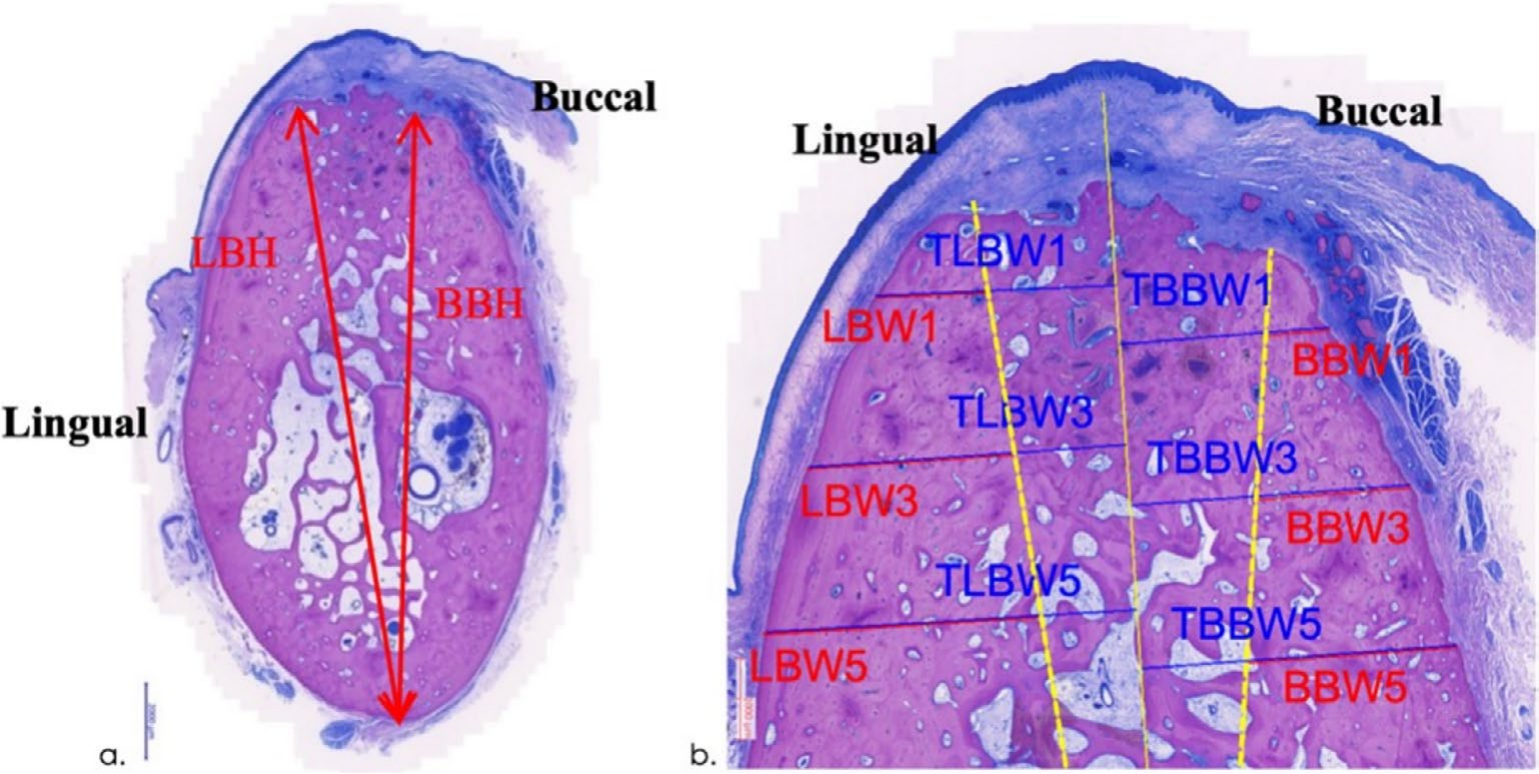
Landmarks defined for the histomorphometrical analysis (McNeal tetrachrome staining). (a) BBH: Buccal bone height; LBH: Lingual bone height (magnification bar: 2000 μm). (b) BBW/LBW: Buccal/lingual alveolar bone wall. TBBW/TLBW: Total buccal/lingual alveolar bone wall width (magnification bar: 1000 μm).

**FIGURE 3:**
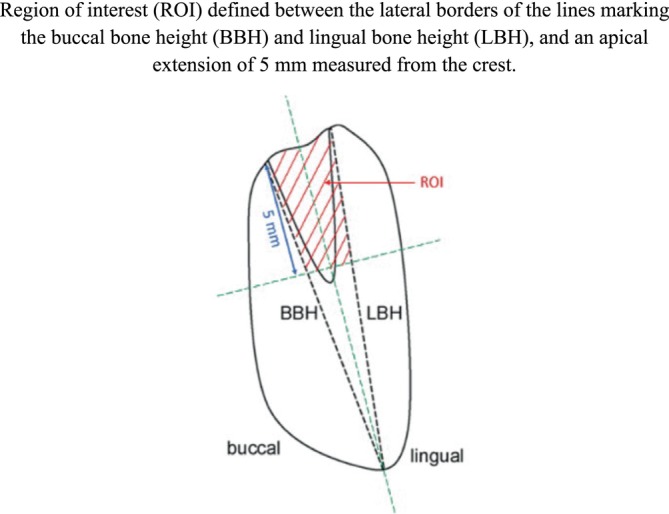
Area measurements (mm^2^) were conducted within a region of interest (ROI) defined between BBH and LBH (lateral borders) and an apical extension of 5 mm measured from the crest.

For each experimental site, the most central section was scanned (Zeiss AXIOSCAN Zl, x20) and analysed with a colour image analysing system (Tribvn, France, CALOPIX 3.2.0) to perform a semi‐automatic analysis of the following parameters (Rothamel et al. 2008) (Figures 2 and 3):

Primary outcome:

1. Buccal Bone Height (BBH) as Measured From the Mandibular Basis to the Respective Buccal Bone Crest (Figure 2a)

Secondary outcomes:

1. Lingual bone height (LBH) as measured from the mandibular basis to the respective lingual bone crest.
2. Buccal and lingual alveolar bone wall width measured from the respective outer cortical bone to BBH or LBH at 1, 3 and 5 mm infracrestally (BBWl, BBW3, BBW5 and LBWl, LBW3, LBW5) (Figure 2b).
3. Total buccal and lingual bone width measured from the respective outer cortical bone to the centre of the ridge at 1, 3 and 5 mm (TBBWl, TBBW3, TBBW5 and TLBWl, TLBW3, TLBW5) (Figure 2b).
4. Area measurements (mm^2^) of the bone and particle surface, fibrous tissue surface and bone marrow surface within the region of interest between BBH and LBH (lateral borders) and an apical extension of 5 mm measured from the crest (Figure 3).

### 2.10 Statistical Analysis

The statistical analysis was accomplished using a commercially available software program (IBM SPSS Statistics 24.0, IBM Corp., Armonk, NY, USA) by NAMSA. The animal served as the statistical unit. The histomorphometrical measurements were performed on one of the most central sections per treated site.

Mean values and standard deviations were calculated for all parameters. Between‐group comparisons were accomplished using ANOVA and Mann– Whitney tests and Bonferroni correction at 0.3%. The alpha error was set at 0.05.

## 3 Results

### 3.1 Clinical Observations

Postoperative wound healing was uneventful in all animals. No dehiscences were noted at any of the test and control sites investigated.

### 3.2 Qualitative Histological Analysis

**FIGURE 4:**
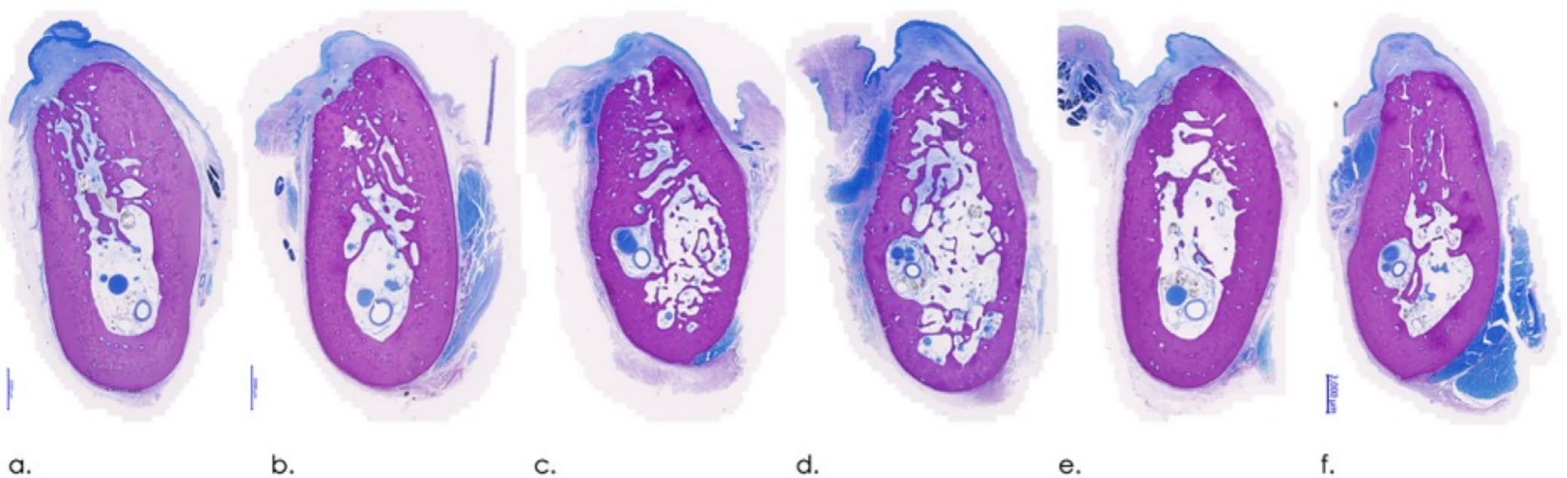
Representative histological views in different groups at 12 weeks (buccal—left; McNeal tetrachrome staining, magnification bar: 2000 μm). (a) N group. (b) C group. (c) T1 group. (d) T2 group. (e) T3 group. (f) T4 group.

Representative histological views of wound healing in different groups are depicted in Figure 4.

At all sites investigated, the overlying soft tissue compartment revealed no marked inflammatory cell infiltrates and showed no histological signs of dehiscences (Figure 4a). A residual root fragment was noted at one C site, which was not observed during surgery.

**FIGURE 5:**
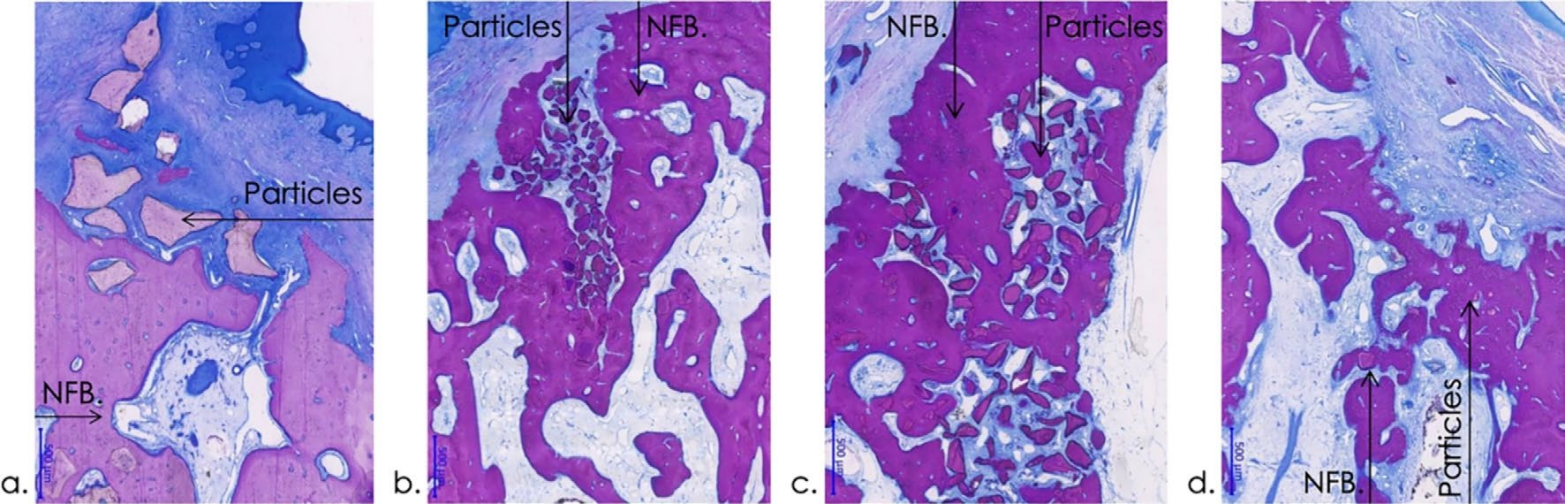
Representative higher magnification histological views in different groups at 12 weeks (magnification bar: 500 μm, NBF: New bone formation). (a) Scattered particles were commonly encapsulated in the lamina propria and occasionally in the alveolar ridge crest (T4 group). (b) Particles in the extraction socket showed significant osteoconduction and osteointegration. (c) Moderate bone marrow formation and osteoblast activity were observed, with slight material degradation, fibroplasia and bone remodelling, leading to variable osteonal formation (T3 group). (d) In all groups, slight resorptive changes (osteolysis) were present primarily (e) observed on the buccal aspects (T4 group).

A few dispersed C, T1, T2, T3 and T4 particles were encapsulated and surrounded by phagocytes and/or pre‐osteoblasts in the dense fibrous layer of the lamina propria (C) and occasionally in the most crestal compartment of the alveolar ridge (Figures 4b and 5a). Within the former extraction socket area, C, T1, T2, T3 and T4 particles were associated with a marked grade of osteoconduction and osteointegration (Figure 5b,c). A cell‐mediated material degradation of a slight grade was commonly noted. Bone marrow was moderately generated in the alveolus along with a slight to moderate fibroplasia. The bone trabeculae commonly harboured a moderate to marked number of active osteoblasts. Slight to moderate signs of bone remodelling resulting in rare (C, T1, T3) to frequent (T2, T4) osteonal formation were noted in the upper part of the sites. Resorptional changes of a slight grade (osteolysis) were predominantly noted at the buccal aspects (Figures 4d–f and 5d).

A complete crestal ridge closure was observed in a total of four out of nine N sites, three out of nine C sites, three out of nine T1 sites, three out of nine T2 sites, seven out of eight T3 sites, and one out of nine T4 sites, respectively.

### 3.3 Histomorphometrical Analysis of Linear Measurements

Mean linear measurements of BBH, BBW, TBB, LBH, LBW and TLBW values are summarised in Tables 1 and 2.

**TABLE 1:** Linear histomorphometrical measurements at the buccal aspect.

Group		BBH	BBW1	TBBW1	BBW3	TBBW3	BBW5	TBBW5
N (*n* = 9)	Mean	14.2	0.6	2.1	1.2	2.4	1.8	2.8
	SD	1.5	0.1	0.1	0.2	0.1	0.3	0.2
C (*n* = 9)	Mean	14.3	0.6	2.2	1.2	2.6	1.7	2.8
	SD	1.2	0.2	0.3	0.3	0.5	0.5	0.6
T1 (*n* = 9)	Mean	14.1	0.7	2.2	1.2	2.5	1.9	3.0
	SD	0.7	0.3	0.2	0.3	0.2	0.5	0.5
T2 (*n* = 9)	Mean	14.0	0.7	2.3	1.3	2.6	2.0	3.0
	SD	1.3	0.2	0.3	0.3	0.4	0.4	0.4
T3 (*n* = 9)	Mean	13.7	0.7	2.3	1.3	2.7	2.1	3.3
	SD	1.3	0.2	0.3	0.4	0.4	0.5	0.5
T4 (*n* = 9)	Mean	13.8	0.6	2.2	1.4	2.7	2.1	3.1
	SD	1.0	0.2	0.3	0.5	0.6	0.6	0.7

*Note:* N—negative control group, C—control group, SD—standard deviation, T1–T4—test groups, BBH‐ buccal bone height, BBW1—buccal alveolar bone wall width measured 1 mm infracrestally, BBW3—buccal alveolar bone wall width measured 3 mm infracrestally, BBW5—buccal alveolar bone wall width measured 1 mm infracrestally, TBBW1—total buccal bone width measured 1 mm infracrestally, TBBW3—total buccal bone width measured 3 mm infracrestally, TBBW5—total buccal bone width measured 5 mm infracrestally. Between‐group differences did not reach any statistical significance in the parameters investigated (*p* > 0.05, respectively).

**TABLE 2:** Linear histomorphometrical measurements at the lingual aspect.

Group		LBH	LBW1	TLBW1	LBW3	TLBW3	LBW5	TLBW5
N (*n* = 9)	Mean	16.5	1.5	3.1	2.5	3.9	3.2	4.4
	SD	1.0	0.4	0.5	0.6	0.7	0.7	0.8
C (*n* = 9)	Mean	16.3	1.2	2.9	2.3	3.8	3.1	4.3
	SD	0.4	0.3	0.4	0.4	0.5	0.4	0.5
T1 (*n* = 9)	Mean	16.3	1.4	3.0	2.3	3.8	3.0	4.3
	SD	0.8	0.2	0.3	0.3	0.5	0.5	0.6
T2 (*n* = 9)	Mean	16.3	1.3	3.0	2.3	3.8	3.1	4.1
	SD	0.5	0.3	0.5	0.5	0.7	0.6	1.0
T3 (*n* = 9)	Mean	16.0	1.1	3.1	2.1	3.9	2.8	4.3
	SD	0.8	0.2	0.4	0.4	0.5	0.5	0.6
T4 (*n* = 9)	Mean	16.4	1.4	3.1	2.4	3.9	3.1	4.3
	SD	0.6	0.3	0.3	0.4	0.4	0.4	0.4

*Note:* N—negative control group, C—control group, SD—standard deviation, T1–T4—test groups, LBH‐ lingual bone height, LBW1—lingual alveolar bone wall width measured 1 mm infracrestally, LBW3—lingual alveolar bone wall width measured 3 mm infracrestally, LBW5—lingual alveolar bone wall width measured 1 mm infracrestally, TLBW1—total lingual bone width measured 1 mm infracrestally, TLBW3—total lingual bone width measured 3 mm infracrestally, TLBW5—total lingual bone width measured 5 mm infracrestally. Between‐group differences did not reach any statistical significance in the parameters investigated (*p* > 0.05, respectively).

All test and control groups were associated with similar BBH and LBH values. In particular, among the T1–T4 groups, BBH scores ranged from 13.7 ± 1.3 mm (T3 groups) to 14.1 ± 0.7 mm (T1 group). In the C and N groups, the corresponding values were 14.3 ± 1.2 mm and 1.4 ± 1.5 mm, respectively. The LBH values were commonly higher in all groups and ranged from 16.0 ± 0.8 mm (T3 group) to 16.0 ± 0.6 mm (T4 group) among the T1–T4 groups. The corresponding values in the C and N groups were 16.3 ± 0.4 mm (C group) and 16.5 ± 1.0 mm (N group). The between‐group differences in BBH and LBH values did not reach any statistical significance (*p* > 0.05, respectively) (Tables 1 and 2).

In all groups, BBW values were smallest at 1 mm and increased towards the 3 and 5 mm reference points below the crest. In particular, among the T1–T4 groups, mean BBW1 values varied between 0.6 ± 0.2 mm (T4 group) and 0.7 ± 0.3 mm (T1 group). In the C and N groups, BBW1 values were 0.6 ± 0.2 mm and 0.6 ± 0.2 mm, respectively. The highest values were noted for BBW5 values, ranging between 1.9 ± 0.5 mm (T1 group) and 2.1 ± 0.6 mm (T4 group) among the T1–T4 groups (Table 1). The respective values in the C and N groups were 1.7 ± 0.5 mm (C group) and 1.8 ± 0.3 mm (N group).

Likewise, LBW values were also smallest at 1 mm, with mean LBW1 values ranging from 1.1 ± 0.2 mm (T4 group) and 1.4 ± 0.3 mm (T4 group) to 1.1 ± 0.2 mm (T3 group) among the T1–T4 groups. The respective measurements in the C and N groups were 1.2 ± 0.3 mm (G group) and 1.5 ± 0.4 mm (N group). In all groups, LBW values commonly increased in the apical direction, resulting in LBW3 values across the T1–T4 groups ranging from 2.1 ± 0.4 mm (T3 group) to 2.4 ± 0.4 mm (T4 group). The respective values in the C and N groups were 2.3 ± 04 mm (C group) and 2.5 ± 0.6 mm (N group). The LBW5 values in the T1–T4 groups varied between 2.8 ± 0.5 mm (T3 group) and 3.1 ± 0.6 mm (T2 group), and they measured 3.1 ± 0.6 mm and 3.1 ± 0.4 mm in the C and N groups, respectively (Table 2). The between‐group differences in BBW and LBW values did not reach any statistical significance (*p* > 0.05, respectively).

Similar outcomes were also noted for both TBBW and TLBW values. In particular, mean TBBW1‐5 values were commonly smaller than the corresponding TLBW1‐5 values, and both TBBW and TLBW measurements increased in the apical direction. The between‐group differences in TBBW1‐5 and TLBW1‐5 values did not reach any statistical significance (*p* > 0.05, respectively) (Tables 1 and 2).

### 3.4 Histomorphometrical Analysis of Area Measurements

The surface area measurements (mm^2^) and the corresponding percentages are presented in Tables 3 and 4. The mean scores for bone and particles at grafted sites among the T1–T4 groups ranged from 9.1 ± 2.1 mm^2^ (52.8% ± 9.8%) (T4 group) to 31.1 ± 2.8 mm^2^ (66.3% ± 10.0%) (T2 group), and it was 11.5 ± 3.7 mm^2^ (61.1% ± 11.4%) in the C group. The respective values tended to be lowest in the N group, amounting to 9.1 ± 2.1 mm^2^ (52.8% ± 9.8%).

**TABLE 3:** Surface area measurements (mm^2^).

Group		Bone and particles	Fibrous tissue	Bone marrow	Artefacts^a^	Supra‐crestal‐free bone and particles	Total area
N (*n* = 9)	Mean	12.5	6.2	0.8	0.4	0.2	20.1
	SD	3.0	2.9	1.0	0.4	0.3	5.0
C (*n* = 9)	Mean	11.5	6.2	0.4	0.3	0.0	18.3
	SD	3.7	1.2	0.5	0.5	0.0	3.3
T1 (*n* = 9)	Mean	11.6	6.7	0.4	0.1	0.0	18.9
	SD	3.0	2.4	0.4	0.2	0.0	4.6
T2 (*n* = 9)	Mean	13.1	6.2	0.7	0.0	0.0	20.0
	SD	2.8	2.6	0.4	0.1	0.0	4.3
T3 (*n* = 9)	Mean	11.9	6.1	1.6	0.2	0.0	19.9
	SD	2.7	2.6	1.8	0.3	0.0	4.5
T4 (*n* = 9)	Mean	9.1	6.9	0.7	0.5	0.0	17.3
	SD	2.1	1.6	0.8	0.9	0.0	2.4

*Note:* N—negative control group, C—control group, SD—standard deviation, T1–T4—test groups. Between group differences did not reach any statistical significances in the parameters investigated (*p* > 0.05, respectively).

^a^ Histological artefact or residual root.

**TABLE 4:** Surface area measurements (%).

Group		Bone and particles	Fibrous tissue	Bone marrow	Artefacts^a^	Supra‐crestal‐free bone and particles
N (*n* = 9)	Mean	62.8	30.1	3.7	2.0	1.3
	SD	1.0	8.5	4.9	2.3	1.6
C (*n* = 9)	Mean	61.1	35.2	2.0	1.7	0.0
	SD	11.4	11.7	2.3	2.9	0.0
T1 (*n* = 9)	Mean	61.8	35.1	2.1	1.0	0.0
	SD	8.4	7.6	2.1	1.3	0.0
T2 (*n* = 9)	Mean	66.3	29.9	3.6	0.1	0.0
	SD	10.0	10.3	2.4	0.3	0.0
T3 (*n* = 9)	Mean	60.7	30.8	7.2	1.3	0.0
	SD	9.7	10.1	6.9	1.8	0.0
T4 (*n* = 9)	Mean	52.8	40.0	4.1	3.2	0.0
	SD	9.8	7.6	4.9	5.9	0.0

*Note:* N—negative control group, C—control group, SD—standard deviation, T1–T4—test groups. Between group differences did not reach any statistical significances in the parameters investigated (*p* > 0.05, respectively).

^a^ Histological artefact or residual root.

The fibrous tissue surface among the T1–T4 groups ranged between 6.1 ± 2.6 mm^2^ (30.8% ± 10.1%) (T3 group) and 6.9 ± 1.6 mm^2^ (40.0% ± 7.6%) (T4 group). In the C and N groups, the respective values were 6.2 ± 1.2 mm^2^ (35.2% ± 11.7%) and 6.2 ± 2.9 mm^2^ (30.1% ± 8.5%), respectively. Mean values for the bone marrow surface were heterogeneous and ranged from 0.4 ± 0.4 mm^2^ (2.1% ± 2.1%) (T1 group) to 1.6 ± 1.8 mm^2^ (7.2% ± 6.9%) (T3 group) among the T1–T4 groups and amounted to 0.4 ± 0.5 mm^2^ (2.0% ± 2.3%) in the C group and 0.8 ± 1.0 mm^2^ (3.7% ± 4.9%). The between‐group differences did not reach any statistical significance in the outcomes assessed (*p* > 0.05, respectively) (Tables 3 and 4).

## 4 Discussion

The present pre‐clinical study aimed at evaluating the performance of four injectable bone fillers (i.e., T1–T4) for ARP in an established animal model (M. G. Araujo et al. 2010; M. G. Araujo and Lindhe 2009). Basically, the histomorphometrical analysis at 12 weeks revealed that mean linear measurements of buccal and lingual bone wall heights and widths were similar in all test and control groups investigated. Moreover, it was noted that at all sites investigated bone width and height measurements on the lingual aspect were commonly higher than the corresponding values measured on the buccal aspect. Likewise, buccal and lingual bone width measurements were smallest at the 1 mm reference points and increased in the apical direction. All these findings corroborate previous data on the alveolar ridge dimensions following tooth extraction in the canine (M. G. Araujo and Lindhe 2005).

When further interpreting the results of the present analysis, it must be realised that these are the first data on T1–T4 materials and published data on injectable bone fillers used for ARP in the canine are very limited (Boix et al. 2006; Rothamel et al. 2008). In particular, a nanocrystalline hydroxyapatite paste also failed to reveal any significant differences in dimensional alveolar ridge alterations at 3 and 6 months when compared with untreated control sites. Notably, this material was associated with poor osteoconductive properties within the extraction socket of material resorption (Rothamel et al. 2008). In contrast, an injectable polymer solution containing biphasic calcium phosphate particles was associated with a better preservation of the alveolar ridge dimensions at 3 months in both the maxilla and mandible (Boix et al. 2006).

Previous studies also reported on the comparison of the use of particulated bone filler and spontaneous healing groups in a similar animal model (M. Araujo et al. 2008; M. G. Araujo et al. 2010; M. G. Araujo and Lindhe 2009; Fickl et al. 2008). In particular, over a period of 6 months, sites treated with particulated bone filler had no apparent effect on the remodelling of the extraction socket, but resulted in a better preservation of the ridge contour when compared with sites left for spontaneous healing (M. Araujo et al. 2008; M. G. Araujo and Lindhe 2009; Fickl et al. 2008).

The discrepancy noted between the aforementioned studies and the present results may be attributed to significant differences in the methods used for assessing alveolar ridge dimensions. Techniques such as using casts and scanning with superimposition at three time points (Fickl et al. 2008) are more sensitive for detecting subtle superficial changes in alveolar contour with minimal alterations. Additionally, histomorphometric analyses for evaluating alveolar ridge changes can vary greatly among studies due to factors such as employing relative percentages for defect‐filling areas or limiting assessments to grafted versus non‐grafted sites (M. Araujo et al. 2008; M. G. Araujo and Lindhe 2009). However, these methods often lack detailed compartmentalisation of the grafted area, which is essential for a histological evaluation of material interactions at different levels of the alveolar bone. It is also worth noting that the present analysis focused on a single healing time point (12 weeks), precluding a multi‐time‐point or long‐term evaluation of dimensional changes. Previous studies have demonstrated differences emerging at 24 weeks (M. G. Araujo and Lindhe 2009).

When further evaluating the present qualitative histological analysis, it was noted that all test and control materials did not fail any inflammatory cell infiltrates in the adjacent soft tissue compartment. Furthermore, C, T1, T2, T3 and T4 particles were associated with a similar, marked grade of osteoconduction and osteointegration within the former extraction socket area, as evidenced by trabecular bone and osteonal formation. These findings basically corroborate the histological characteristics of bone particles incorporation following ARP, as observed over a period of 4 weeks in the canine (M. G. Araujo et al. 2010) and humans (M. G. Araujo et al. 2015). After 4 months following the ARP human, the deproteinized bovine bone mineral particles were integrated with the newly formed host bone and retained the volume of the hard tissue defect (M. G. Araujo et al. 2015). In fact, as shown by one recent histological analysis on human biopsies, the longer healing time after ARP was associated with an increased incorporation of collagenated bovine bone mineral particles (Couso‐Queiruga et al. 2023). More specifically, after 12, 24 and 36 weeks, the proportion of mineralized tissue increased (13.53%, 33.33% and 37.05%), whereas the proportion of non‐mineralized tissue (63.32%, 55.55% and 52.48%) and remaining xenograft material (16.94%, 10.69% and 9.46%) decreased over time (Couso‐Queiruga et al. 2023).

Notably, the present area measurements also confirmed a partial persistence of bovine bone particles used for T1–T4 grafts, which was associated with a trend for higher mean bone surface values when compared with the N group. Even though these differences did not reach statistical significance, the potential effect of the resulting lower surface areas of fibrous tissue at grafted sites on the osseointegration of dental implants needs to be further investigated.

Moreover, it must be emphasised that a potential limitation of the present analysis was the lack of any soft‐tissue‐related measurements, including the assessment of a preservation of the soft tissue contour, which should also be addressed in future studies. Finally, in the present experimental study, the efficiency of novel bone augmentation materials has been tested in standardised defects in order to allow for a quantitative analysis, which, however, does not reflect the clinical reality (e.g., compromised extraction sockets) and thus may affect the regenerative potential. As such, the present findings must be further validated in future clinical studies.

As shown by one former experimental study, the compressive forces exerted on a particulate graft material during ARP have a direct effect on bone regeneration in the apical region (Delgado‐Ruiz et al. 2018). In particular, histomorphometrical analysis revealed a significantly higher amount of residual bone filler particles in the apical region of an extraction socket treated with a force of 200 g, compared with those treated with a force of 10 and 50 g (Delgado‐Ruiz et al. 2018). After 2 months, the percentage of a newly formed bone in the apical region was considerably higher in the group treated with higher forces (i.e., 200 g) compared with those treated with the lower compressive forces (i.e., 10 or 50 g). The latter finding clearly suggests that the higher compressive forces facilitate the penetration of the particulated graft material into the apical area of the extraction socket and lead to enhanced bone formation at the apical third. In this context, it might be speculated that the use of an injectable bone filler may not only ease the clinical handling but also lead to enhanced fill of the defect that is not dependent upon the compressive forces being applied.

Within its limitations, the present study has pointed to the similar efficacy of injectable bone fillers in terms of the preservation of alveolar ridge dimensions compared with the particulated bone filler and the negative control.

## Author Contributions

Conceptualization: Frank Schwarz (lead) and Puria Parvini (supporting). Data collection and analysis: Frank Schwarz, Ausra Ramanauskaite, Karina Obreja, Jonas Lorenz, Puria Parvini. Writing – original draft: Frank Schwarz (lead). Writing – review and editing: Ausra Ramanauskaite, Karina Obreja, Jonas Lorenz, Puria Parvini.

## Conflicts of Interest

The authors declare no conflicts of interest.

## Supporting information


**Table S1.** Allocation of test and control groups (*n* = 9 animals).

## Data Availability

The data that support the findings of this study are available on request from the corresponding author. The data are not publicly available due to privacy or ethical restrictions.
