# Supplementary material for: Efficacy of Injectable Bone Fillers for Alveolar Ridge Preservation: A Histomorphometrical Analysis
Source: J Clin Periodontol. 2025 Apr 10;52(7):949–59. doi: 10.1111/jcpe.14162 (PMC12176456; doi:10.1111/jcpe.14162)
Supplement: Supplementary file 1 — Table S1. Allocation of test and control groups (n = 9 animals). [file JCPE-52-949-s001.docx]

**Supplement - Tables**

***Supplement Table 1*.** Allocation of test and control groups (n=9 animals).

| **Time-Period** | **Animal No.** | **Left hemi-mandible** | | | **Right hemi-mandible** | | |
| --- | --- | --- | --- | --- | --- | --- | --- |
|  |  | **P2** | **P3** | **P4** | **P2** | **P3** | **P4** |
| 12 weeks | 752 | N | C | T3 | T2 | T4 | T1 |
|  | 324 | T1 | N | T4 | C | T2 | T3 |
|  | 388 | T4 | C | N | T2 | T1 | T3 |
|  | 617 | T2 | T3 | N | T1 | C | T4 |
|  | 381 | T3 | T4 | C | N | T2 | T1 |
|  | 921 | C | T2 | T1 | T3 | T4 | N |
|  | 587 | T1 | N | T2 | T4 | T3 | C |
|  | 612 | N | T1 | T2 | C | T3 | T4 |
|  | 726 | T4 | T1 | C | T3 | N | T2 |

T1: Test group 1: M2L (mixed with blood)

T2: Test group 2: M2L (mixed with saline)

T3: Test group 3: M2rtu

T4: Test group 4: M4L (mixed with saline)

C: Control Group: Bio-Oss Collagen

N: Negative control: Spontaneous Healing
